# Supplementary material for: GPs’ motivation for teaching medical students in a rural area—development of the Motivation for Medical Education Questionnaire (MoME-Q)
Source: PeerJ. 2019 Jan 24;7:e6235. doi: 10.7717/peerj.6235 (PMC6348089; doi:10.7717/peerj.6235)
Supplement: Table S1 [file peerj-07-6235-s001.docx]

**Supplementary Table 1.** Initial Version of the MoME-Questionnaire (28 items)

|  |  | agree | slightly agree | slightly not agree | not agree |
| --- | --- | --- | --- | --- | --- |
| 1 | I want to contribute in promoting and educating medical students. | 1 | 2 | 3 | 4 |
| 2 | It is my social responsibility to actively participate in the education of medical students. | 1 | 2 | 3 | 4 |
| 3 | I have a mind to educate students and to share my knowledge. | 1 | 2 | 3 | 4 |
| 4 | Educating students is a knowledge exchange where both sides may benefit from. | 1 | 2 | 3 | 4 |
| 5 | Being an „Academic Teaching Practice“ (related to a university) enhances the status of my practice. | 1 | 2 | 3 | 4 |
| 6 | Patients feel that I am more qualified if future medical doctors are trained in my practice. | 1 | 2 | 3 | 4 |
| 7 | Being an „Academic Teaching Practice“ (related to a university) is publicity for my practice. | 1 | 2 | 3 | 4 |
| *8* | *I hope to attract more patients being an „Academic Teaching Practice“.** | *1* | *2* | *3* | *4* |
| 9 | I hope that cooperating with a university facilitates access to evidence based information. | 1 | 2 | 3 | 4 |
| 10 | Cooperating with the university is a good chance to get touch with colleagues and build a network. | 1 | 2 | 3 | 4 |
| 11 | Cooperating with the university increases my chances to find a successor form my own practice. | 1 | 2 | 3 | 4 |
|  |  | agree | slightly agree | slightly not agree | not agree |
| *12* | *Students can support and relieve me in daily routine patient care.“ ** | *1* | *2* | *3* | *4* |
| 13 | Students can spend more time with patients what increases patients’ satisfaction. | 1 | 2 | 3 | 4 |
| 14 | Teaching students also means to be up-to-date with respect to medical information. | 1 | 2 | 3 | 4 |
| *15* | *I hope that „General Practice“ gets more attention if more GPs take part in medical education of students.** | *1* | *2* | *3* | *4* |
| 16 | Positive experiences I made during my own training period motivate me to participate in the education of medical students. | 1 | 2 | 3 | 4 |
| 17 | Negative experiences I made during my own training period motivate me to participate in the education of medical students. | 1 | 2 | 3 | 4 |
| 18 | I believe I am to old to teach medical students. | 1 | 2 | 3 | 4 |
| 19 | I do not have sufficient didactical compretencies. | 1 | 2 | 3 | 4 |
| 20 | Students derange practice administration. | 1 | 2 | 3 | 4 |
| 21 | I can treat less patients if I instruct students in my practice. | 1 | 2 | 3 | 4 |
| 22 | Being exposed to students frequently my patients are less satisfied. | 1 | 2 | 3 | 4 |
| 23 | I operate at full capacity regarding patient treatment. This is why I do not have time to teach and train students **in my practice.** | 1 | 2 | 3 | 4 |
|  |  |  |  |  |  |
|  |  | agree | slightly agree | slightly not agree | not agree |
| 24 | I operate at full capacity regarding patient treatment. This is why I do not have time to teach and train students **out of my practice.** | 1 | 2 | 3 | 4 |
| *25* | *I have made bad experiences with medical students in my practice in the past** | *1* | *2* | *3* | *4* |
| 26 | I am not interested in teaching medical students (lectures at the university). | 1 | 2 | 3 | 4 |
| 27 | I am not interested in instructing medical students in my practice. | 1 | 2 | 3 | 4 |
| 28 | Family commitments debar me from participating in teaching students. | 1 | 2 | 3 | 4 |

items belonging to factor „commitment“

items belonging to factor “personal benefit”

*items excluded in the final version of the questionnaire
