# Supplementary material for: GPs’ motivation for teaching medical students in a rural area—development of the Motivation for Medical Education Questionnaire (MoME-Q)
Source: PeerJ. 2019 Jan 24;7:e6235. doi: 10.7717/peerj.6235 (PMC6348089; doi:10.7717/peerj.6235)
Supplement: Table S2 [file peerj-07-6235-s002.docx]

**Supplementary Table 3.** German version of the modified MoME-Q

|  |  | Trifft zu | Trifft eher zu | Trifft eher nicht zu | Trifft nicht zu |
| --- | --- | --- | --- | --- | --- |
| 1 | Ich möchte einen Beitrag zur Nachwuchsförderung leisten. | 1 | 2 | 3 | 4 |
| 2 | Ich sehe es als meine Verantwortung gegenüber der Gesellschaft an, mich an der studentischen Ausbildung aktiv zu beteiligen. | 1 | 2 | 3 | 4 |
| 3 | Ich habe Lust darauf, Studenten etwas beizubringen und mein Wissen zu teilen. | 1 | 2 | 3 | 4 |
| 4 | Ich sehe studentische Lehre als einen Wissensaustausch, von dem beide Seiten profitieren. | 1 | 2 | 3 | 4 |
| 5 | Mit dem Zusatz „Akademische Lehrpraxis“ wird meine Praxis aufgewertet. | 1 | 2 | 3 | 4 |
| 6 | Patienten nehmen mich als kompetenter wahr, wenn ich in meiner Praxis künftige Mediziner ausbilde. | 1 | 2 | 3 | 4 |
| 7 | Lehrpraxis einer Universität zu sein bedeutet Werbung für meine Praxis | 1 | 2 | 3 | 4 |
| 8 | Ich erhoffe mir über den Kontakt zur Universität einen erleichterten Zugang zu evidenzbasierten Informationen. | 1 | 2 | 3 | 4 |
| 9 | Ich sehe in der Kooperation Chancen, neue Kontakte und Netzwerke zu knüpfen. | 1 | 2 | 3 | 4 |
| 10 | Ich erhöhe durch die Kooperation meine Chance, einen Nachfolger für meine eigene Praxis zu bekommen. | 1 | 2 | 3 | 4 |
| 11 | Studenten können mehr Zeit mit Patienten verbringen, wodurch die Patienten zufriedener sind. | 1 | 2 | 3 | 4 |
|  |  | Trifft zu | Trifft eher zu | Trifft eher nicht zu | Trifft nicht zu |
| 12 | Studentische Lehre bedeutet für mich, auch selbst auf dem neuesten Stand des medizinischen Wissens bleiben zu können. | 1 | 2 | 3 | 4 |
| 13 | Eigene Positiverfahrungen aus meiner eigenen Ausbildungszeit sind für mich Motivation, mich an der studentischen Lehre zu beteiligen. | 1 | 2 | 3 | 4 |
| 14 | Eigene Negativerfahrungen aus meiner eigenen Ausbildungszeit sind für mich Motivation, mich an der studentischen Lehre zu beteiligen. | 1 | 2 | 3 | 4 |
| 15 | Ich halte mich für zu alt, um Studenten etwas beibringen zu können. | 1 | 2 | 3 | 4 |
| 16 | Ich verfüge nicht über ausreichende didaktische Fähigkeiten. | 1 | 2 | 3 | 4 |
| 17 | Studenten stören den Praxisablauf. | 1 | 2 | 3 | 4 |
| 18 | Wenn ich Studierende in der Praxis anleite, kann ich weniger Patienten versorgen. | 1 | 2 | 3 | 4 |
| 19 | Durch häufigen Kontakt mit Studierenden sind meine Patienten weniger zufrieden mit der Behandlung. | 1 | 2 | 3 | 4 |
| 20 | Ich bin mit der Patientenversorgung derart ausgelastet, dass ich für studentische Lehre **in der Praxis** keine Zeit aufbringen kann. | 1 | 2 | 3 | 4 |
| 21 | Ich bin mit der Patientenversorgung derart ausgelastet, dass ich für studentische Lehre **außerhalb meiner Praxis** keine Zeit aufbringen kann. | 1 | 2 | 3 | 4 |
| 22 | Ich habe kein Interesse an studentischer Lehre (Theorie). | 1 | 2 | 3 | 4 |
| 23 | Ich habe kein Interesse an der Anleitung der Studierenden (Praxis). | 1 | 2 | 3 | 4 |
| 24 | Familiäre Verpflichtungen hindern mich daran, mich an studentischer Lehre zu beteiligen. | 1 | 2 | 3 | 4 |

Items des Faktors „Engagement“

Items des Faktors „Persönliche Vorteile“
